# Supplementary material for: Inhibition of Notch Signaling Enhances Antitumor Activity of Histone Deacetylase Inhibitor LAQ824
Source: Int J Mol Sci. 2023 Sep 4;24(17):13660. doi: 10.3390/ijms241713660 (PMC10487749; doi:10.3390/ijms241713660)
Supplement: Supplementary file 1 [file ijms-24-13660-s001.zip › Figure S1.pdf]

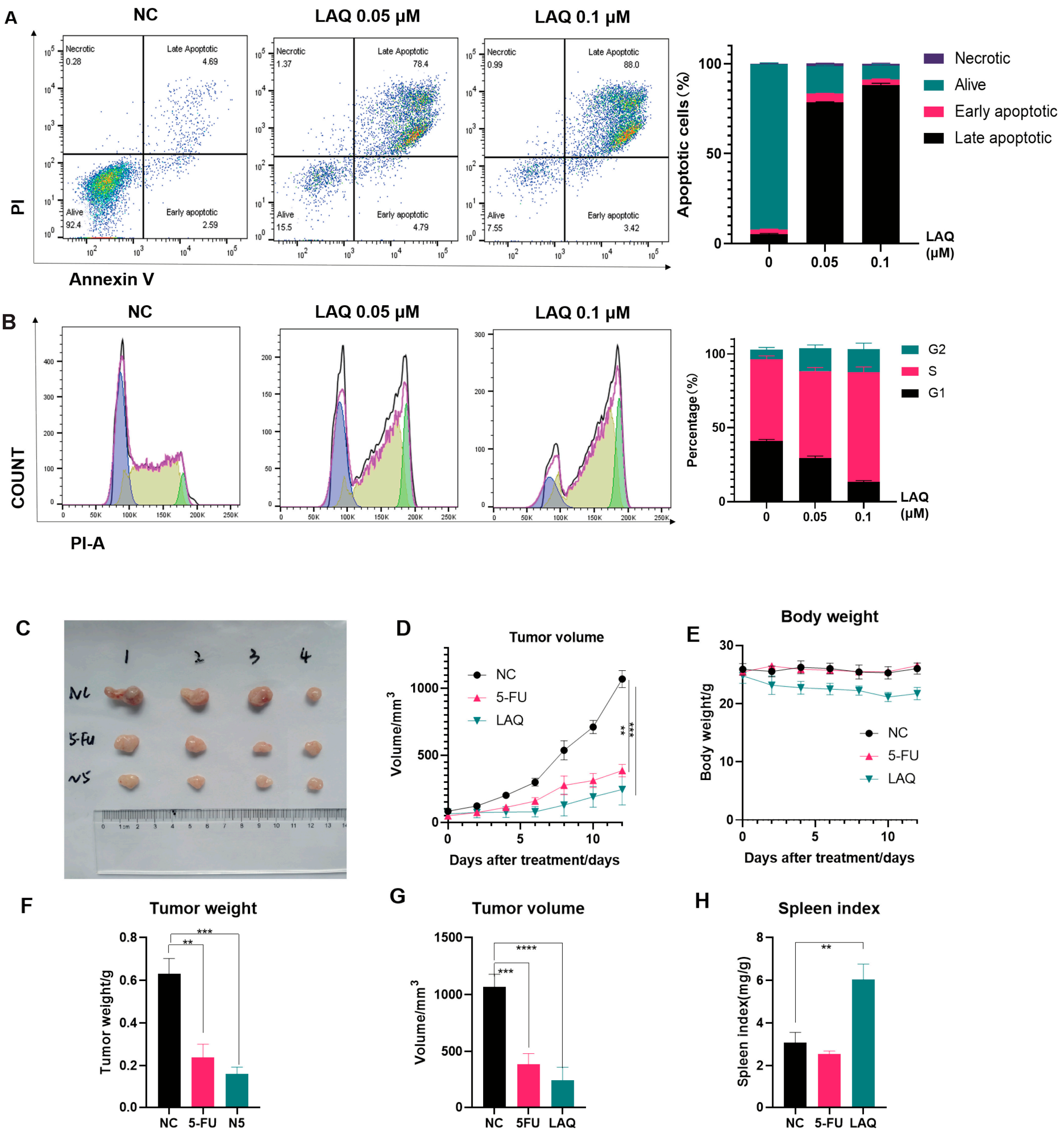

Figure S1: LAQ inhibits tumor growth in vitro and in vivo (A) Apoptosis (annexin V+/DAPI− plus annexin V+/DAPI+) of HCT116 cell line treated with different concentrations of LAQ was detected by flow cytometry. (B) HCT116 cell line were treated with different concentrations of LAQ for 24 h, then fixed, stained with PI, and analyzed by flow cytometry. (C) Photographic records of the tumors in each group described above that were removed on day 14. (D) Recording of the body weight in each group during gavage. (E) Expression of the tumor volume in each group during gavage. (F) (G) Illustration of the tumors volume and tumor weight in each group described above that were removed on day 14. (H) Recording of spleen index in each group. The data are expressed as the mean ± SD. Significant differences were analyzed using the unpaired two-tailed *t*-test. * $p < 0.05$, ** $p < 0.01$, *** $p < 0.001$, **** $p < 0.0001$. n=4.
