# Supplementary material for: Inhibition of Notch Signaling Enhances Antitumor Activity of Histone Deacetylase Inhibitor LAQ824
Source: Int J Mol Sci. 2023 Sep 4;24(17):13660. doi: 10.3390/ijms241713660 (PMC10487749; doi:10.3390/ijms241713660)
Supplement: Supplementary file 1 [file ijms-24-13660-s001.zip › Figure S2.pdf]

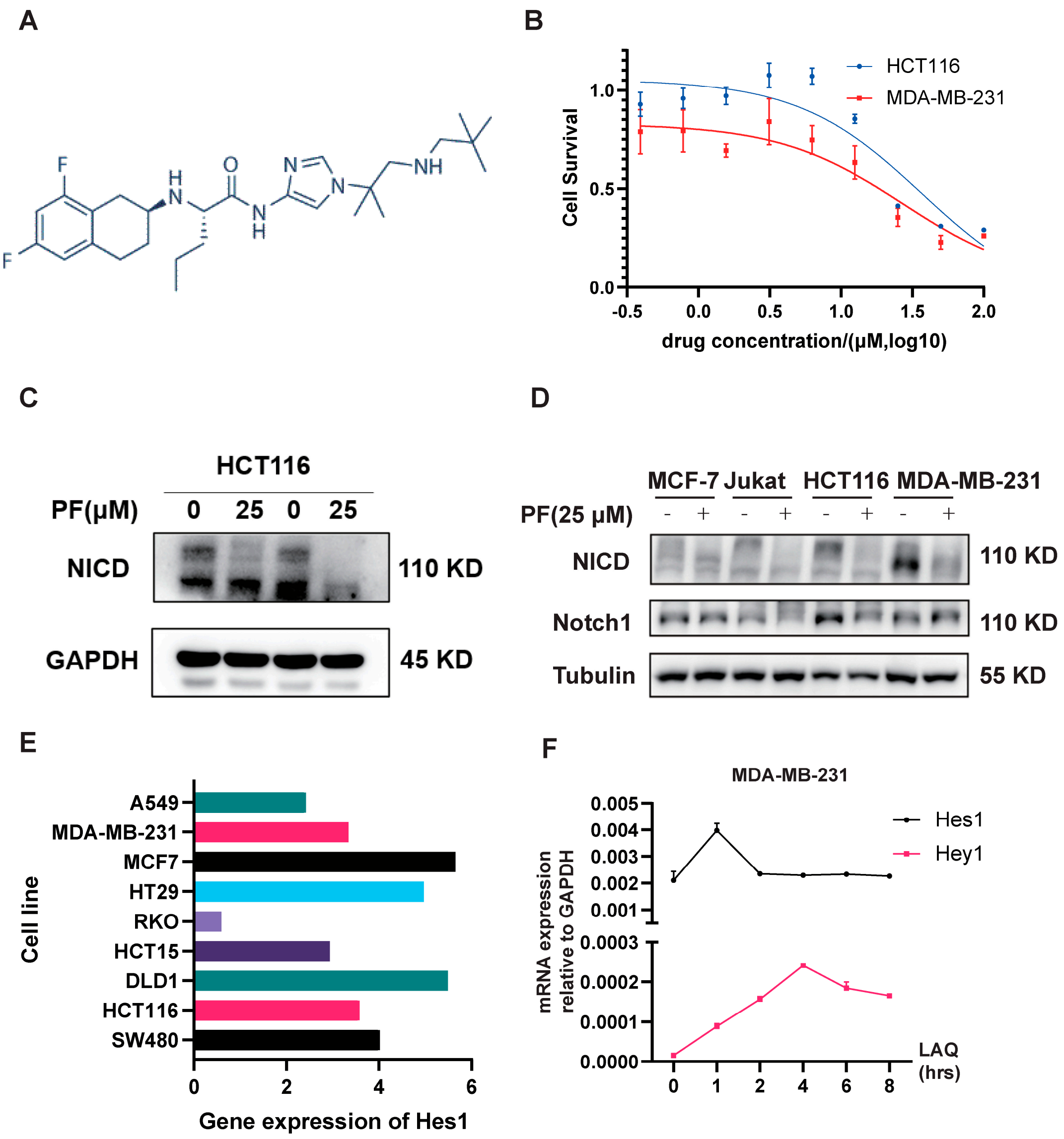

G

Cell line	IC50（μM）
SW480	32.21±0.401
DLD-1	> 100
HCT-15	34.61±0.3514
RKO	> 100
HT-29	> 100
HCT116	0.006593±0.8088
MDA-MB-231	29.29±1.414
MCF7	>100
A549	0.01003±1.076

Figure S2: Effect of the Notch signaling inhibitor PF in tumor cell lines （A）The structre of PF03084014. (B) MTT assay detects the IC50 of PF in HCT116 and MDA-MB-231 cell lines. (C) Western blotting analysis of protein NICD treated with PF in HCT116 cell line. (D) The expression of NICD and Notch1 were detected by Western blotting. (E) We screened out Hes1 in 9 solid tumor cell lines from the genomics of drug sensitivity in the cancer database and found that Hes1 was high expression

in MDA-MB-231 cell line. (F) The expression of Hes1 and Hey1 were detected by qPCR after treatment with LAQ for different time in MDA-MB-231 cell line. (G) The table of IC50 values for LAQ in different 9 cell lines. The data are expressed as the mean ± SD. Significant differences were analyzed using the unpaired two-tailed *t*-test. * $p < 0.05$, *** $p < 0.001$. n=3.
